# Environmental Remediation of Arsenate-Contaminated Groundwater Using a Graphene Oxide-Supported Cu-NPs/UiO-66(Zr)-NH_2_ Nanocomposite

**DOI:** 10.3390/nano16080462

**Published:** 2026-04-14

**Authors:** Faten M. Ali Zainy, Doaa S. Al-Raimi, Amr A. Yakout

**Affiliations:** Department of Chemistry, College of Science, University of Jeddah, P.O. Box 80237, Jeddah 21589, Saudi Arabia; fmzainy@uj.edu.sa (F.M.A.Z.);

**Keywords:** arsenate As(V), UiO-66-NH_2_, kinetics, adsorption, graphene oxide

## Abstract

Arsenic contamination, mainly in the arsenate (As(V)) form, continues to pose a serious threat to groundwater quality worldwide due to its long-term stability and toxicity at very low levels. Herein, we demonstrate, for the first time, a three-dimensional graphene oxide-based nanocomposite composed of Cu nanoparticle-doped, amino-functionalized UiO-66 (Cu/UiO-66-NH_2_) anchored on a graphene oxide framework (Cu/UiO-66-NH_2_@GO) as a novel and efficient nanosorbent for the rapid removal of As(V) in groundwater-like solutions. The nanocomposite was characterized by SEM and HRTEM to confirm the hybrid structure and by XRD, N_2_ adsorption–desorption isotherms, and XPS to investigate crystallinity, porosity, and surface chemistry. The derived material exhibited a highly dispersed morphology and performed rapid arsenate solid-phase extraction to attain equilibration within 10 min and was effective for a wide pH range of 2–11. The best fit for the kinetic profiles was provided by the pseudo-second-order model. Interestingly, the maximum adsorption capacity of 747.9 mg g^−1^ at pH 6.8 was achieved, demonstrating the benefits of the complementary pairing of dispersive GO sheets and Zr-MOF adsorption domains with Cu-derived active sites. Mechanistically, the enhanced uptake is ascribed to a combination of effects, including electrostatic pre-concentration, ligand exchange, and inner-sphere complexation at metal-oxo nodes; spectroscopic analysis (XPS and FTIR) suggests that the majority of arsenate is immobilized via a strong Zr-O-As bond at coordinatively unsaturated Zr centers, which is in line with t-ZrO_2_-like surface domains formed within the nanocomposite. The embedded GO support inhibits further framework interpenetration and enhances active site availability and mass transport, leading to fast and high-capacity arsenate capture in groundwater samples with related conditions. Taken together, this work presents a powerful design concept that integrates unique GO-supported, Cu-modified UiO-66-NH_2_ with Zr-O binding motifs to afford high-rate remediation nanocomposites, providing an excellent platform for next-generation arsenate remediation materials.

## 1. Introduction

Arsenic in groundwater and industrial wastewater is still a serious concern because even small amounts can harm human health over time [1]. In water, it is mainly present as arsenite [As(III)] and arsenate [As(V)], with arsenate usually being the more common form under normal environmental conditions [1,2]. Long-term exposure has been linked to arsenicosis and several cancers, including those of the bladder, lung, kidney, and skin. Because of these risks, the World Health Organization set the safe limit for arsenic in drinking water at 10 μg L^−1^, yet levels above this value are still reported in many parts of the world, including Mexico, Central Italy, parts of China, Bangladesh, and India [2,3,4,5,6]. This makes the search for dependable treatment methods especially important.

Several techniques have been used to remove arsenic from water, such as coagulation, oxidation, membrane separation, ion exchange, and adsorption [7]. Among them, adsorption is often favored because it is easier to operate, relatively inexpensive, and offers high removal efficiency [7,8]. Many adsorbents have been tested for this purpose, including activated carbon, carbon nanotubes, graphene-based materials, zeolites, resins, metal oxides, and metal–organic frameworks [9,10]. Zirconia (ZrO_2_) has attracted particular interest because it is stable in water, has low toxicity, and shows good affinity for arsenic species [11]. Still, ordinary ZrO_2_ nanoparticles are not without problems; they often show limited capacity, slow uptake, and a tendency to aggregate, all of which reduce their practical performance [12,13]. Similar drawbacks are also seen in several widely used sorbents, especially when treatment is carried out in real groundwater systems containing competing ions and varying pH [14,15]. For that reason, recent work has focused on developing improved Zr-based adsorbents that combine strong arsenic binding with faster kinetics, better dispersion, and greater stability under realistic conditions.

Metal–organic frameworks (MOFs) have emerged as a new class of porous crystalline materials featuring a high surface area, tunable pore environment, and modular chemistry which have been extensively explored in adsorption [16,17,18,19], separation [20,21], gas storage [22], catalysis [23], sensing, and drug delivery [24,25]. However, applying MOFs to aqueous adsorption is still infrequent and challenging, mainly due to some frameworks exhibiting slow uptake kinetics and/or particle aggregation, and/or limited stability in aqueous media [26,27,28]. Significant attempts have therefore been made in the direction of the synthesis of water-stable MOFs, namely, zeolitic imidazolate frameworks (ZIFs) and zirconium-based UiO systems. UiO-66, made from Zr^4+^ nodes and terephthalic acid linkers, exhibits resistance to acidic and basic conditions and has been investigated for pollutant elimination. In addition, the adsorption kinetics, separation efficiency, and recyclability can be improved by polymerizing UiO-66 with carbon nanotubes, GO, or magnetic materials [29]. Performance of UiO-66 in As removal: Following the successful adsorption of Pb(II), pristine UiO-66 has also been reported in the literature for the removal of As(III) by many researchers. They reported an As(V) adsorption capacity of ~90 mg g^−1^, which is due to the superior adsorption affinity between arsenate and Zr–OH pockets [30]. Yet its practical application is still hindered due to the agglomeration and limited dispersion of the catalyst as well as mass transfer restriction, which can retard adsorption and complicate the separation of solids and liquids.

To enhance affinity and promote fast As(V) uptake, Zr-based MOFs have been adapted in terms of functionalization and structural modulation. Reported capacities for UiO-66 are up to approximately 303 mg g^−1^ at pH 2 and ∼148 mg g^−1^ at neutral pH, stemming from potentially strong coordination interactions between arsenate and Zr-OH sites as well as possible hydrogen bonding with the linker environment. Functional derivatives yield even higher performance: the amino-functionalized UiO-66-NH_2_ adsorbs ∼77 mg g^−1^ at pH 7.0, and its capacity can be raised to approximately 161 mg g^−1^ after acid treatment that protonates –NH_2_ groups [31,32,33]. In addition to amino functionalities, post-synthetic ligand exchange, defect engineering, and the introduction of functionalities like -SO3H or –OH can be applied to boost the density and accessibility of the active sites, while compositing with silica, iron oxides, or carbon-based supports can enhance mechanical stability, dispersibility, and adsorption kinetics [34,35,36,37]. Zr-based materials, however, are generally considered to possess a robust affinity to arsenic over a wide pH range, further underscoring the potential of Zr-based adsorption chemistry for real-water applications [38].

However, it is still difficult to separate nanoscale UiO-66-based adsorbents and they easily become irregularly shaped and agglomerated, which decreases the accessible surface and inhibits fast pollutant capturing [39]. Therefore, matrix-supported growth and core–shell engineering have been developed to manipulate particle dispersion and morphology, with silica as a common choice for mechanically robust support. In fact, amino-functionalized UiO-66-NH_2_ has been proven to be efficient for CO_2_/N_2_ separation and fluoride adsorption, which is in line with the strong electron-donating effect of –NH_2_ groups that potentially increase anion absorption through electrostatic and/or specific interactions. Audu et al. showed that UiO-66(Zr)-NH_2_ adsorbed approximately 10% more phosphate than UiO-66 [40]. Since phosphate and arsenate are isostructural and possess similar Brønsted basicity in water [41], sorbents with high affinity for phosphate are usually considered to show strong affinity for arsenate. Motivated by these considerations, structured UiO-66-NH_2_ nanocomposites that suppress aggregation and enhance kinetics have been engineered, including core–shell microspheres in which regulated MOF shell growth can lead to improved microporosity and site accessibility as well as straightforward separation.

In this work, we report that it is possible to go beyond the usual concept of silica-supported UiO-66-NH_2_-based architectures and develop a GO-supported Cu/UiO-66-NH_2_ nanocomposite for the fast removal of arsenate from groundwater samples. The novelty lies in the combination of (i) a high-aspect-ratio GO template that prevents MOF particle agglomeration and retains aqueous dispersibility, (ii) amino-functionalized UiO-66 adsorption units that provide strong Zr-based binding motifs for As(V), and (iii) Cu-based functionalities that introduce additional reactive sites, which may catalyze joint uptake routes. By combining these features into a single hybrid system, it is possible that the system will combine a high site density with effective mass transport and easy separability, all of which are essential for managing complex groundwater matrices. Hence, in this study, we report the synthesis of the GO-supported Cu/UiO-66-NH_2_ nanocomposite and its comprehensive investigation for arsenate adsorption, including physicochemical characterization, adsorption capacity, uptake kinetics, and pH dependence, along with understanding the mechanistic basis of its superior performance under groundwater-like environments. The novelty of this work lies in the rational design of a GO-supported Cu/UiO-66-NH_2_ nanocomposite, which integrates metal functionalization with conductive support to enhance active site accessibility. This engineered system demonstrates superior arsenate removal in groundwater, along with faster adsorption kinetics and improved stability compared to the parent materials.

## 2. Experimental Work

### 2.1. Chemicals and Reagents

All chemicals and reagents were of analytical grade and used as received. Zirconium(IV) chloride (ZrCl_4_), 2-aminoterephthalic acid (NH_2_–H_2_BDC), *N*,*N*-dimethylformamide (DMF), acetic acid, and acetonitrile (ACN) were obtained from J&K Scientific (Beijing, China). Copper(II) chloride trihydrate and sodium hydroxide of analytical-reagent quality were used. Graphene oxide (GO) powder was supplied by Sinopharm Chemical Reagent Co., Ltd. (Shanghai, China). A certified arsenate standard solution (H_3_AsO_4_, 1000 mg L^−1^) was obtained from Millipore (Darmstadt, Germany).

### 2.2. Instrumentation

Concentrations of arsenate solutions were determined using a UVD-3500 UV-Vis spectrophotometer operating at a wavelength of 190–950 nm. High-resolution transmission electron microscopy (HRTEM; JEOL JEM-2100V, Tokyo, Japan) and scanning electron microscopy (SEM; JEOL JSM-6010LV, Japan) were used to study the morphology and microstructure of the Cu/UiO-66-NH_2_@GO nanocomposite. X-ray photoelectron spectroscopy (XPS; Thermo ESCALAB 250Xi, Waltham, MA, USA) was used to investigate the oxidation state and surface elemental composition. Using a Rigaku D/MAX-2550 diffractometer with Cu Kα radiation, powder X-ray diffraction (XRD) was used to assess phase analysis and crystallinity. Surface functional groups were identified using Fourier-transform infrared spectroscopy (FTIR; Nicolet 400) from 4000 to 400 cm^−1^. Using a Zetasizer Nano ZS90 (Malvern, UK), the zeta potential of a 1 weight percent aqueous dispersion of the nanocomposite was determined. Using a calibrated Fisher Scientific pH meter (Model 810, Waltham, MA, USA) with a combination glass electrode, the pH of the solution was determined.

### 2.3. Synthesis of Cu/UiO-66-NH_2_@GO Nanocomposite

The Cu/UiO-66-NH_2_@GO nanocomposite was synthesized by combining Cu-NPs with a UiO-66-NH_2_ layer deposited on GO and then subjecting the mixture to solvent-free mechanical treatment in a ball-milling reactor. In brief, UiO-66-NH_2_ was solvothermally synthesized in the presence of GO nanosheets, and the obtained UiO-66-NH_2_@GO solid was then mixed with pre-synthesized Cu-NPs by high-energy milling. To synthesize UiO-66-NH_2_@GO, 1.0 g of GO was dispersed in 60 mL DMF in a three-neck flask by brief sonication, followed by magnetic stirring for 15 min. Simultaneously, 1.0 g of ZrCl_4_ was dissolved in 60 mL DMF and added to the GO dispersion under stirring; the mixture was stirred for 1 h at room temperature [42,43]. Subsequently, 230 mg of NH_2_–H_2_BDC was dissolved in 60 mL DMF containing 1.5 mL of acetic acid and added to the reaction flask. The mixture was then heated at 120 °C with continuous stirring for 24 h. After cooling to room temperature, the product was harvested by centrifugation (6000 rpm, 5 min) and washed several times with DMF and ethanol. The solid was then dried in a vacuum oven at 80 °C for 24 h and activated in a vacuum at 190 °C for 12 h. The Cu-NPs were prepared hydrothermally in a stainless-steel autoclave with CuCl_2_ as the copper source and L-ascorbic acid as the reducing agent, and the pH of the reaction mixture was adjusted to 11 with 0.1 M NaOH [44]. Finally, the Cu-NPs were loaded into UiO-66-NH_2_@GO by mechanically mixing the solids and ball-milling for 30 min at 25 Hz to produce the Cu/UiO-66-NH_2_@GO nanocomposite.

### 2.4. Batch Adsorption Experiments

Batch adsorption tests were conducted in capped centrifuge tubes. Unless otherwise stated, 10 mg of Cu/UiO-66-NH_2_@GO was contacted with 10 mL of As(V) solution (initial concentration, 10–100 mg L^−1^). The suspension was briefly sonicated (~1 min) to ensure uniform dispersion, and the solution pH was adjusted to 7.8 using 0.1 mol L^−1^ HCl or NaOH. The tubes were agitated in a thermostatic orbital shaker at 250 rpm and 25 °C. At predetermined times, the solid was separated, and the supernatant was filtered through a 0.45 µm membrane (22 mm) prior to analysis. Residual As(V) was quantified by atomic absorption spectrometry (AAS; Vario-6, Analytik Jena, Jena, Germany) using external calibration prepared from standards of known As(V) concentrations; the instrumental detection limit was 0.5 µg L^−1^.

The influence of pH on adsorption was assessed by adjusting the initial pH of the As(V) solutions over pH 2–11. Kinetic experiments were performed by sampling aliquots at time intervals between 1 and 60 min to evaluate adsorption rates. Equilibrium isotherms were obtained at 25 °C by varying the initial As(V) concentration from 10 to 100 mg L^−1^ under otherwise identical conditions. Control experiments were conducted using UiO-66-NH_2_ as the adsorbent under the same procedure for comparison.

Removal efficiency and adsorption capacity were calculated according to Equations (1) and (2), respectively:

(1) $$\%\;R=\;\frac{C_{o}-\;C_{t}\;}{c_{o}}\;\times \;100$$

(2) $$\%\;q_{t}=\frac{{(C}_{o}-C_{t})\;V\;}{m}\;$$

where *C_o_* and *C*_*t*_ (mg L^−1^) are the initial As(V) concentrations and at time *t*, *V* (L) is the solution volume, and *m* (g) is the mass of nanocomposite.

All experiments were performed in triplicate and reported as mean values. Method precision was evaluated using four replicates (*n* = 4) by calculating percentage recovery and relative standard deviation (RSD). Matrix effects were examined by comparing slopes of matrix-matched and aqueous calibration curves using one-way ANOVA.

## 3. Results and Discussion

### 3.1. Characterization of Cu/UiO-66-NH_2_@GO Nanocomposite

**Figure 1:**
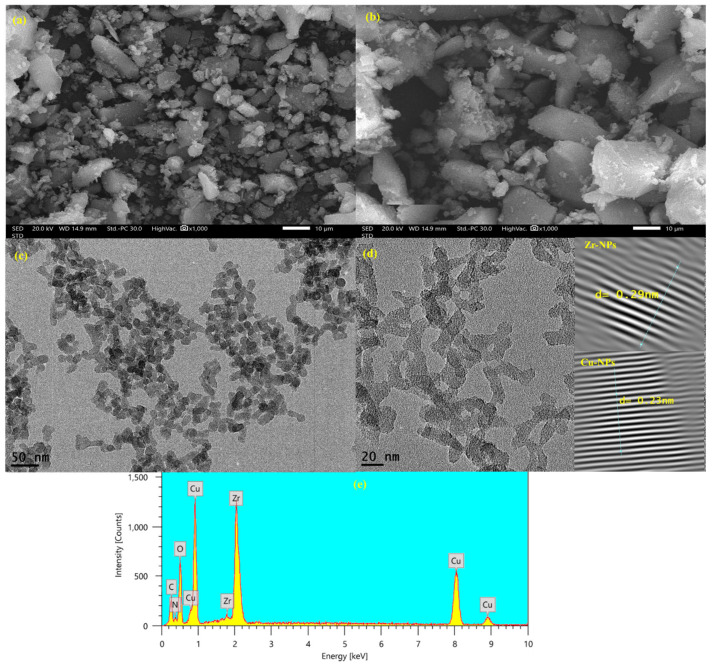
SEM (**a**,**b**), HRTEM (**c**,**d**) and EDX (**e**) of Cu/UiO-66-NH_2_@GO.

The surface morphology (SEM and HRTEM) of the Cu-NPs/NH_2_-UiO-66(Zr)/GO nanocomposite is presented in Figure 1. As seen in the SEM images (Figure 1a,b), the surface of the nanocomposite is quite rough and has a high intensity of microparticles. This suggests the firm bonding between the Cu NPs and UiO-66(Zr) moiety and the GO matrix rather than producing a smooth and detached phase. The TEM pictures (Figure 1c,d) show a high density of nanoscale domains over thin GO/MOF layers, which suggests strong interfacial contact between the components and good dispersion of the nanoparticles. Distinct d-spacings of around 0.23 and 0.29 nm are apparent in the HRTEM lattice fringes (right panel), and they are ascribed to Cu- and Zr-based nanodomains, respectively. This suggests also the existence of crystalline phases as opposed to amorphous deposits. Finally, the EDS spectrum (Figure 1e) shows high Zr and Cu peaks in addition to high C and O peaks (GO framework), which clearly indicate that Cu-NPs were successfully loaded onto the Zr-MOF/GO matrix. Briefly, the micro-rough surface with evenly spaced nanoparticle morphology is very beneficial for adsorption because it promotes contact between the Cu/Zr-functional interfaces and As(V) ions, and this increases the number of active sites.

**Figure 2:**
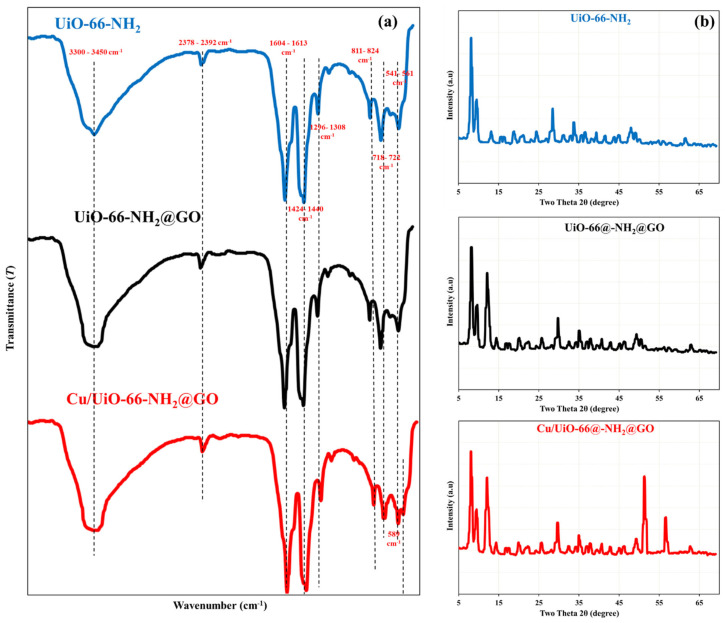
FTIR (**a**) and XRD (**b**) of Cu/UiO-66-NH_2_@GO.

The FTIR spectra (Figure 2a) of UiO-66-NH_2_, UiO-66-NH_2_@GO, and Cu/UiO-66-NH_2_@GO offer further information regarding nanocomposite structure and As(V) binding. O-H/N-H stretching vibrations are responsible for the broad peak at 3300–3450 cm^−1^ for UiO-66-NH_2_, while the asymmetric and symmetric stretching modes of the coordinated carboxylate groups in the organic linker are responsible for the peaks at 1604–1613 cm^−1^ and 1424–1440 cm^−1^ [45]. The peak at 1296–1308 cm^−1^ is attributed to the amino group’s C–N stretching, confirming the existence of –NH_2_ groups. The integrity of the metal–organic framework is confirmed by the presence of the Zr-O characteristic peaks in the lower wavenumber range (541–561 cm^−1^) [46]. The O-H and C=O peaks somewhat broaden and vary in strength after GO is added, confirming the hydrogen bonding and interface interactions between UiO-66 (Zr)-NH_2_ and the oxygenated groups of GO.

After Cu loading, the appearance of the band at 589 cm^−1^ can be attributed to Cu-O bonds, which verifies the successful anchoring of Cu species on the nanocomposite. After As(V) adsorption, the obvious changes in the O-H/N-H and carboxylate regions reveal the involvement of hydroxyl, amino, and metal–oxygen functional groups in As(V) adsorption. The above spectral features are in line with the surface complexation model between arsenate oxyanions and Zr-OH/Cu-OH active sites, assisted by hydrogen bonding and electrostatic forces, which highlights the cooperative effect of the MOF structure, GO sheets, and Cu species in the efficient removal of As(V).

The UiO-66-NH_2_, UiO-66-NH_2_@GO, and Cu/UiO-66-NH_2_@GO XRD patterns (Figure 2b) show that the nanocomposite was successfully synthesized and that its structural integrity held up well during the arsenate removal procedure. Sharp peaks at low 2θ values (about 7–9° and 12–13°) in the virgin UiO-66-NH_2_ diffraction pattern are characteristic of the highly crystalline Zr-based MOF structure and agree with the face-centered cubic structure of UiO-66 that has been described [47,48]. The primary diffraction peaks of UiO-66-NH_2_ are maintained, with a slight drop in strength with the addition of GO, indicating that the MOF’s crystallinity is mainly preserved while it is evenly distributed over the GO sheets. For GO, the absence of a noticeable peak at about 10–11° suggests effective exfoliation and even dispersion throughout the nanocomposite. The diffraction pattern maintains the distinctive peaks of UiO-66-NH_2_ after Cu loading, suggesting that the structural integrity is preserved. Cu can be attributed to the faint peaks at high 2θ values, which show successful loading without the formation of massive crystallites.

**Figure 3:**
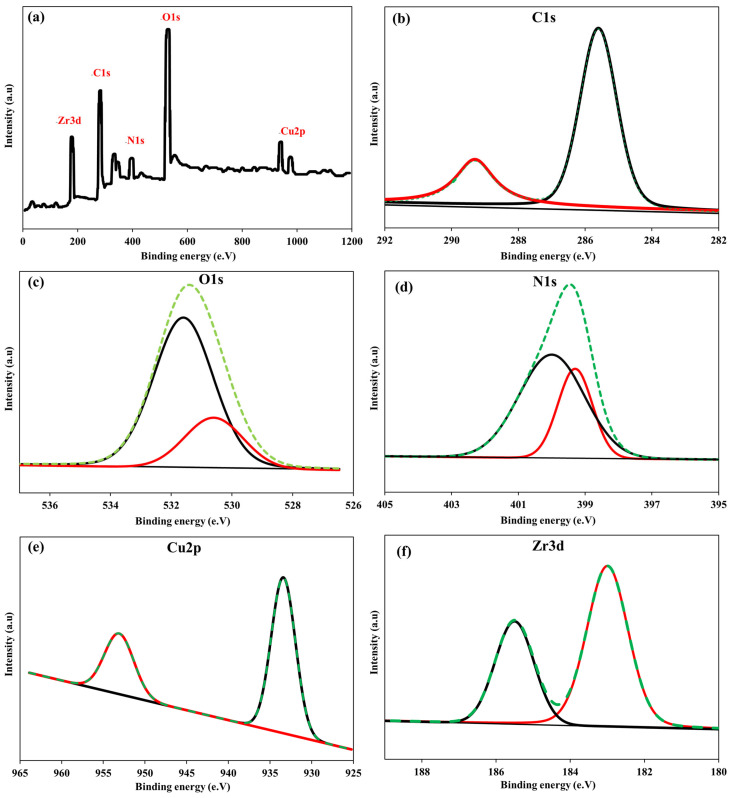
Full survey (**a**) and elemental XPS (**b**–**f**) of Cu/UiO-66-NH_2_@GO nanocomposite.

The XPS spectra (Figure 3) provide conclusive evidence for the successful interaction between As(V) species and the Cu/UiO-66-NH_2_@GO nanocomposite. The survey spectrum (Figure 3a) confirms the presence of C, N, O, Cu, and Zr elements, as expected for the nanocomposite material. High-resolution C1s spectra (Figure 3b) consist of a strong peak at approximately 284.8 eV, which can be ascribed to C–C/C=C bonds, and a shoulder peak around 288–289 eV, which can be ascribed to C=O/O–C=O bonds, suggesting the presence of oxygenated groups of GO and the organic linker of UiO-66-NH_2_ [49]. The N1s spectrum (Figure 3c) consists of peaks around ~399–400 eV, which can be ascribed to –NH_2_/–NH– bonds, suggesting that the amino groups are still available for interaction with arsenate species. The metal–oxygen bonds (Zr-O/Cu-O) are represented by a low binding energy peak (~530–531 eV) in the O1s spectrum profile (Figure 3d), while hydroxyl and adsorbed oxygen species are represented by a high binding energy peak (~531–533 eV). The overwhelming presence of Cu(II) ions in surface coordination complexes is shown by the Cu 2p spectra (Figure 3e), which show characteristic Cu 2p_3_/_2_ and Cu 2p_1_/_2_ peaks with less noticeable satellite structures [50]. Similarly, the Zr3d doublet at about 182–186 eV shows that the UiO-66 framework structure [51] was preserved throughout the adsorption procedure (Figure 3f).

It should be noted that significant surface complexation, not physisorption, is what is indicated by the binding energy values with slight variations and peak intensity changes following As(V) adsorption. When combined, these results highlight the cooperative contributions of all nanocomposites and offer compelling evidence for the dominant role of inner-sphere complexation with Zr-OH and Cu-OH surface sites, along with electrostatic forces and hydrogen bonding interactions with –NH_2_ and oxygen functionalities on GO surfaces.

### 3.2. Impact of pH and Cu/UiO-66-NH_2_@GO Mass Dosage

**Figure 4:**
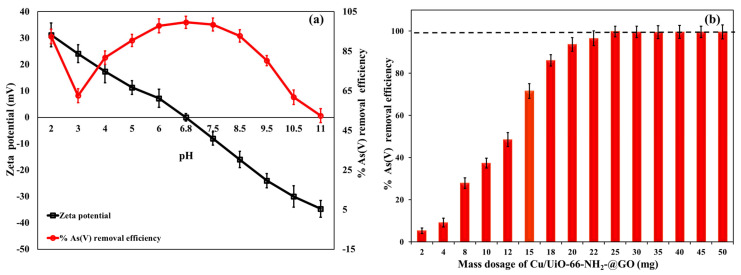
The impact of zeta potential and pH on the adsorptive removal of As(V) (**a**) and mass dosage (**b**) of the Cu/UiO-66-NH_2_@GO nanocomposite at 25.0 °C and 10 min of contact time.

Figure 4a illustrates the strong pH dependence of As(V) removal by Cu/UiO-66-NH_2_@GO, which is consistent with the joint effect of (i) arsenate speciation in solution and (ii) pH-dependent surface chemistry/charge properties of the nanocomposite material. Over the pH range of 2–11, the zeta potential decreases almost linearly from strongly positive values at low pH to more negative values at high pH, passing through the isoelectric point at approximately pH 6.8. Simultaneously, the As(V) removal efficiency varies in a non-linear fashion: high removal efficiency at pH 2.0, gradually decreasing toward mildly acidic pH values (minimum around pH~5), followed by a sharp increase to reach a maximum at near-neutral pH values (≈6–7), with high removal efficiency persisting at mildly alkaline pH values, and finally decreasing at higher pH values above 9–10.

Such a phenomenon is in line with the acid–base equilibria of arsenic acid (H_3_AsO_4_), where the dominant species in solution change from partially protonated species to oxyanions with increasing pH. In the slightly acidic to neutral pH range, H_2_AsO_4_^−^ is the major species, while in the pH range of ~7 and above, the distribution shifts to favor HAsO_4_^2−^. These negatively charged species strongly interact with Zr-based nodes and hydroxylated metal sites via inner-sphere complexation and ligand exchange, rather than simple electrostatic interactions. UiO-66(Zr)-NH_2_ provides coordinately active Zr-OH/Zr-OH_2_ sites that can readily exchange surface hydroxyl groups with arsenate to produce stable Zr-O-As bonds (commonly bidentate/bridging structures), while the Cu nanoparticles provide additional Lewis-acidic/hydroxylated Cu sites that can engage in surface complexation.

At very low pH (≈2), the surface of the nanocomposite is highly positively charged, and arsenate is predominantly H_2_AsO_4_^2−^ with some less dissociated H_3_AsO_4_^−^. Notwithstanding the high proton activity (which, in many cases, can inhibit anion adsorption by protonating surface hydroxyls), the adsorbent demonstrates high removal efficiency. This suggests that As(V) adsorption is not solely driven by electrostatic interactions; rather, very strong specific interactions, especially coordination/ligand exchange at Zr(IV) sites and Cu-containing sites, are still operative despite the lack of expectation from electrostatic interactions. Moreover, highly acidic environments can also enhance arsenate adsorption by favoring the displacement of surface –OH/–OH_2_ ligands and the formation of inner-sphere complexes at metal sites.

With increasing pH from strongly acidic to mildly acidic pH values (≈3–5), the zeta potential decreases significantly, suggesting increasing deprotonation of surface functional groups and reduced positive surface charge. Under these conditions, electrostatic attraction between the arsenate anion and the surface is reduced, although some Zr–OH groups may remain partially protonated, thus reducing the availability of deprotonated surface sites that are more amenable to ligand exchange. The net effect is a temporary reduction in adsorption efficiency, as expected around the minimum point at pH~5.

An additional pH (Figure 4a) increases towards near-neutral values (pH = 6–7) strongly favors As(V) removal, reaching its maximum efficiency (99.6%) at pH 6.8. From a mechanistic perspective, this pH range offers the best compromise: (i) arsenate is mainly present as H_2_AsO_4_^−^ (highly reactive towards metal-OH centers), (ii) Zr-OH groups are sufficiently deprotonated to favor ligand exchange and the formation of strong Zr-O-As inner-sphere bonds, and (iii) the amine functional group of UiO-66-NH_2_ is partially protonated (-NH_3_^+^), providing an additional source of electrostatic binding sites that can help to increase the local arsenate concentration in the vicinity of reactive Zr/Cu centers. The simultaneous occurrence of maximum removal efficiency and isoelectric point values also indicates that specific complexation plays a dominant role even when the net surface charge approaches neutrality, and arsenate uptake is maximum, suggesting that chemisorption/inner-sphere binding is more important than simple electrostatic adsorption.

In the mildly alkaline region (pH range 7–9), the surface charge becomes more negative, and the arsenate species increasingly favor the divalent HAsO_4_^2−^ form. Notwithstanding the onset of electrostatic repulsion, the removal efficiency remains high, again indicating the dominant role of the strong inner-sphere mechanism at Zr and Cu sites, which can effectively counteract the charge-related repulsions. However, above pH 9–10, the adsorption efficiency drops dramatically. This is expected, as (i) the surface charge is strongly negative, (ii) the arsenate species are more highly charged (and thus more repelled), and (iii) the OH^−^ ions aggressively compete for the Zr/Cu and GO surface coordination sites, thus inhibiting ligand exchange reactions and the availability of metal-OH groups that can be displaced by arsenate. In the alkaline region, the joint action of electrostatic repulsion and hydroxide competition leads to a net decrease in affinity and thus the observed efficiency loss.

In summary, Figure 4a shows that the removal of As(V) by Cu/UiO-66-NH_2_@GO is a process dominated by a combination of surface charge interactions and strong inner-sphere complexation at Zr/Cu centers, with secondary roles played by protonated amines. In terms of optimal pH conditions, the slightly basic range (approximately 6–8) would be most preferable for efficient arsenate removal, although the strong adsorption capacity maintained at pH 2.0 suggests that the material could also be used under acidic conditions if needed.

Figure 4b illustrates the significant increase in the dependence of As(V) removal on the amount of Cu/UiO-66-NH_2_@GO used, as expected for a system where the number of available adsorption/complexation sites increases proportionally with the amount of sorbent added. As the amount of the nanocomposite sorbent was increased from 5 to 25 mg, the removal efficiency increased dramatically from approximately 25.1% to approximately 99.6%, suggesting that, at lower concentrations, the process is mainly limited by the lack of surface area and the number of available reactive sites (Zr–OH/Zr–OH_2_ groups in UiO-66-NH_2_, Cu-associated hydroxylated sites, and oxygenated GO regions). In this regime, the addition of more material translates to an increase in the overall number of binding sites accessible for inner-sphere complexation (Zr–O–As bond formation), ligand exchange, and electrostatically facilitated sorption.

The sharp increase in the gain up to 25.0 mg also indicates that the initial loading of As(V) per unit number of active sites is quite high; therefore, even small increments in the amount of sorbent result in large increments in the percentage of arsenate removed. As the dose is increased closer to 25.0 mg, the adsorption capacity reaches the point at which nearly all of the As(V) present in solution can be removed under the given conditions, thereby driving the system toward a “solute-limited” region in which the concentration of the remaining dissolved arsenate is sufficiently low that further increments in the removal percentage are no longer significant; this is indicated by the plateau region beyond 25.0 mg, where further increments in sorbent amount result in only small increments in removal efficiency.

At higher doses (>25 mg), the principle of diminishing returns can be explained by several mechanisms operating simultaneously. First, as the equilibrium concentration of As(V) is close to its lowest level, further increments in the number of available sites cease to contribute to higher removal, as the force driving mass transfer (concentration gradient) is no longer significant. Second, the presence of excess solids can facilitate interparticle interactions and, to some extent, aggregation of the nanocomposite, especially for samples containing GO, which may result in the screening of a portion of the adsorption sites and a reduction in the specific surface area per unit mass. Third, an increase in the concentration of solids can enhance the viscosity of the slurry and introduce diffusion restrictions, further suppressing the additional effectiveness of the sorbent. Thus, 25.0 mg is an optimal dose for operation, which provides a balance between efficient arsenate removal and material economy and favorable dispersion, and this amount was chosen for the subsequent experiments.

### 3.3. The Effect of Contact Time and Adsorption Kinetics

**Figure 5:**
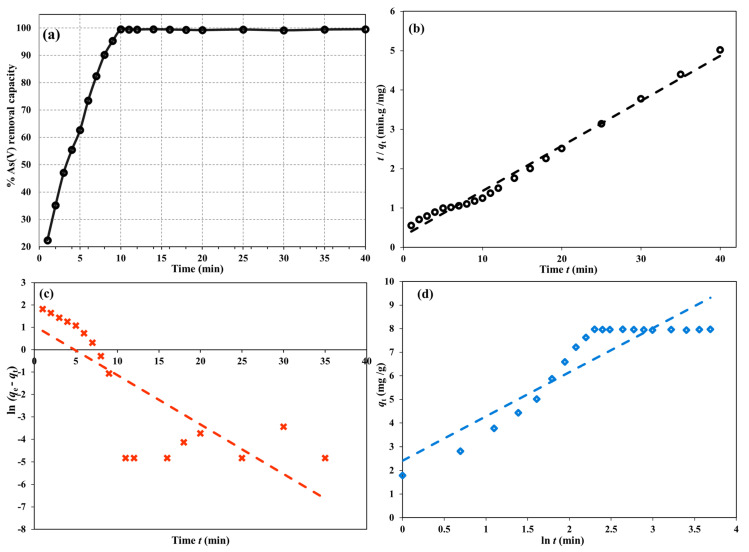
The impact of contact time (**a**). Linear kinetic equations of PSO (**b**), PFO (**c**) and Elovich (**d**) models for the adsorptive removal of As(V) by the Cu/UiO-66-NH2@GO nanocomposite at 25.0 °C, pH 6.5 and a 15 mg mass dose.

The time-dependent adsorption uptake of As(V) onto the Cu/UiO-66-NH_2_@GO nanocomposite (Figure 5a) indicates a very fast adsorption phase, followed by an initial plateau. The removal efficiency quickly occupied the easily accessible active locations, rising from 22.4% at one minute to around 99.6% at 10 min. After 10 min, the plot approached a horizontal line (approximately 99.2–99.6% up to 10 min), further confirming that the system quickly reached the adsorption equilibrium and that only a small fraction of the low-energy sites (or slow intraparticle diffusion processes) is left to control the system’s approach to the final plateau. Under the given experimental conditions (C_0_ = 20 mg/L, V = 10 mL, and m = 25 mg), this plateau indicates an experimental adsorption capacity of approximately 7.97 mg/g, which is in excellent agreement with near-quantitative removal of As(V) in a matter of minutes.

Table 1 presents the findings of the analysis of the experimental results using the pseudo-first-order (PFO), pseudo-second-order (PSO), and Elovich equations (Figure 5b–d) to gain a better understanding of the dominant kinetics [52,53,54]. With a very high correlation coefficient (*R*^2^ ≈ 0.992), the PSO equation produced the best fit of the complete adsorption process (Figure 5b), resulting in an estimated *q*_e_ of 8.74 mg·g^−1^ and k_2_ of 0.045 g·mg^−1^·min^−1^. The excellent agreement with the PSO equation suggests that surface reactions, which are frequently connected to chemisorption contributions, dominate the entire process. It also suggests that the adsorption process entails a rapid surface interaction of As(V) species until the surface sites are saturated. Conversely, the PFO equation resulted in a good fit of the initial adsorption process (Figure 5c) with high linearity for the early data points (*R*^2^ ≈ 0.985, *k*_1_ = 0.208 min^−1^, *q*_e_ = 7.97 mg g^−1^), but it failed when the entire time course was constrained to a single linear relationship, particularly in the vicinity of equilibrium where (*q*_e_ − *q*_t_) is very small and the experimental errors are greatly amplified.

Indeed, when the PFO linearization is carried out using the experimental plateau *q*_e_ and all points, the fit becomes significantly worse (*R*^2^ ≈ 0.638), further supporting the fact that PFO is a proxy for the initial fast uptake process rather than the entire kinetic process. The Elovich equation gave a moderate fit (*R*^2^ ≈ 0.833) with *α* = 6.79 mg·g^−1^·min^−1^ and *β* = 0.534 g·mg^−1^, indicating that the adsorption process (Figure 5d) occurs on an energetically heterogeneous surface where the rate of adsorption exponentially decreases with an increase in surface coverage. Taken together, these results indicate a mechanism where As(V) is removed extremely quickly due to the abundance of high-affinity sites in the initial stage, while the subsequent stage becomes increasingly restricted by site saturation and slower adsorption processes; in any case, the adsorption kinetics are best described by the pseudo-second-order model over the entire time span.

### 3.4. Synergistic Roles of GO, UiO-66-NH_2_, and Cu NPs in Enhancing As(V) Adsorption by Cu/UiO-66-NH_2_@GO

**Figure 6:**
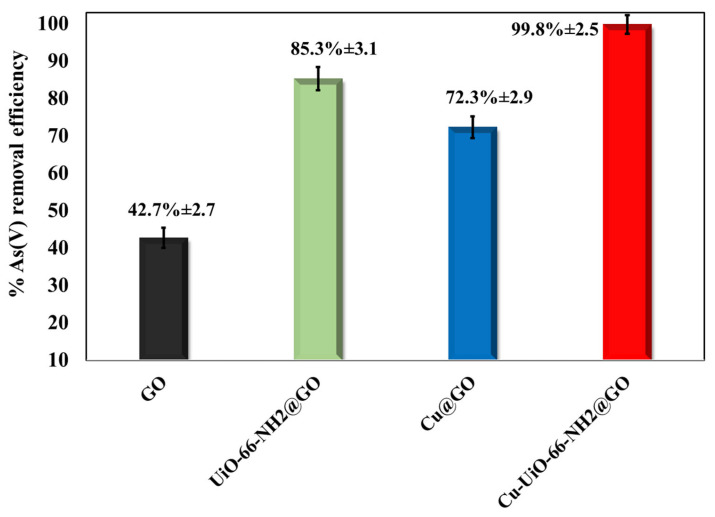
The Cu-NPs and UiO-66(Zr)-NH_2_ nanoparticles’ impact on the Cu/UiO-66-NH_2_@GO nanocomposite’s As(V) adsorptive removal efficiency under the optimum conditions.

The comparative experiment in Figure 6 clearly demonstrates that the synergistic effect of all three components, GO, UiO-66-NH_2_, and Cu-NPs, is responsible for the outstanding As(V) removal ability of Cu/UiO-66-NH_2_@GO, rather than a simple combination of the individual abilities of the three components. The lowest removal efficiency (42.7 ± 2.7%) is observed for pristine GO, which is expected due to the dominance of oxygen-containing functional groups (–COOH/–OH/epoxy) on its surface, which can interact with arsenate ions mainly through weak outer-sphere electrostatic interactions and hydrogen bonding. In addition, at circumneutral pH values, the GO surface is predominantly negatively charged, which repels negatively charged arsenate species (H_2_AsO_4_^−^/HAsO_4_^2−^) and thus inhibits their adsorption.

The addition of UiO-66-NH_2_ to GO leads to a large increase in the amount of arsenate removed (85.3 ± 3.1%), which highlights the significance of Zr nodes and the structure of the MOF. UiO-66-type MOFs exhibit high Lewis acidic Zr(IV) nodes, which are functionalized with Zr-OH/Zr-OH_2_ groups that can specifically coordinate arsenate over inner-sphere complexation and exchange reactions between ligands, therefore leading to the development of stable Zr-O-As bonds. This chemisorption mechanism is much more favorable and irreversible compared to electrostatic interactions and thus leads to a large performance enhancement compared to GO. The enhanced surface polarity and protonatable -NH_2_ groups that result from amino functionalization further boost the arsenate absorption capacity. At mildly acidic to neutral pH, partial protonation of –NH_2_ to –NH_3_^+^ can result in the establishment of local electrostatic “preconcentration” sites for oxyanions around the Zr nodes and can also help in H-bond formation with arsenate, thus facilitating more frequent and efficient binding events at the coordination-active sites.

Cu@GO further enhances As(V) removal efficiency compared to GO (72.3 ± 2.9% for Cu@GO and 42.7 ± 2.75 for GO), thereby indicating that the Cu-based sites are a significant contributing factor, even in the absence of the MOF. In aqueous media, Cu nanoparticles are known to have hydroxylated or partially oxidized surfaces, featuring Cu-OH/Cu-O/CuOOH-type motifs, which can react with arsenate ions either through surface complexation or ion exchange [55,56,57]. These metal–oxygen functional sites can provide stronger active sites for arsenate than GO alone and can also modulate the surface charge and hydration environment in a manner favorable to oxyanion sorption. Moreover, Cu NPs can prevent the restacking of GO layers, consequently maintaining the accessible surface area and facilitating mass transfer.

Even when Cu and UiO-66-NH_2_ are combined on GO, the removal efficiency reaches its maximum value (99.8 ± 2.5%), thus clearly establishing a strong synergistic effect. Several different mechanisms might be working together to establish this synergistic effect. First, GO serves as a conductive and high-surface-area support material that helps to disperse UiO-66-NH_2_ particles and Cu NPs evenly, thus inhibiting their aggregation and increasing the proportion of accessible active sites.

Second, UiO-66-NH_2_ provides a high density of strong Zr-OH inner-sphere binding sites, whereas Cu adds additional hydroxylated/complexation-capable regions so that the resulting nanocomposite shows a heterogeneous surface with multiple binding motifs for As(V) capture for a wider range of microenvironments. Third, the interfacial interaction between Cu and the Zr-MOF (potentially via shared oxygenated bridges or proximity-induced effects on local acidity/basicity) may also facilitate ligand exchange kinetics and arsenate complex stability. Finally, the amine group may facilitate local anionic arsenate enrichment near metal sites, thereby increasing the likelihood of arsenate binding to the strongest sites rather than being confined to the diffuse layer.

While the above component-by-component analysis supports the following functional roles: GO offers a dispersive, high-surface-area support with low intrinsic affinity; UiO-66-NH_2_ offers high-affinity Zr-based inner-sphere binding sites and pH-responsive amine groups to enhance adsorption; and Cu NPs offer additional metal–OH/oxide complexation sites and structural dispersion. Their combination provides a multi-site, highly accessible adsorption interface that explains the near-quantitative As(V) removal achieved with the Cu/UiO-66-NH_2_@GO nanocomposite.

### 3.5. Recycling and Desorption Studies

**Figure 7:**
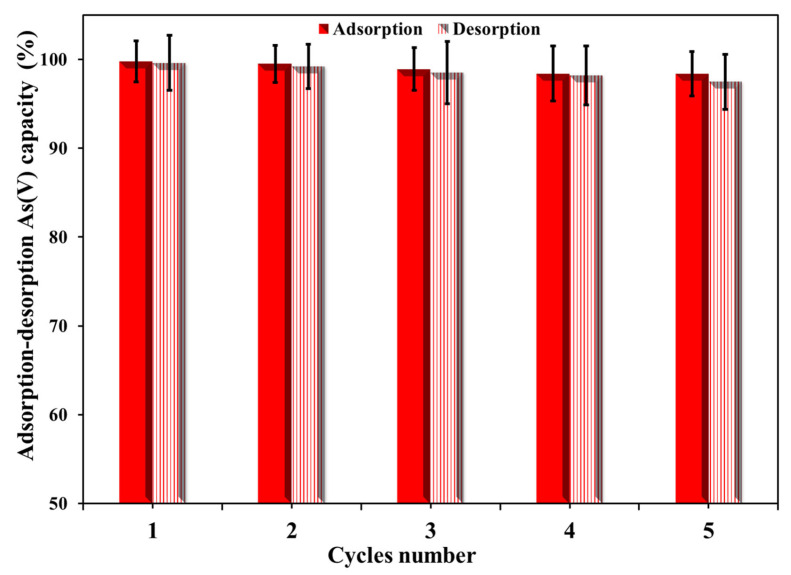
The reusability study for the Cu/UiO-66-NH_2_@GO nanocomposite’s As(V) adsorption–desorption efficiency.

As shown in Figure 7, Cu/UiO-66-NH_2_@GO also exhibits a high level of operational stability in terms of repeated adsorption–desorption cycles, ensuring its applicability in arsenate removal. Under the optimized conditions (10 mL of 20 mg·L^−1^ As(V), pH 6.8, 10 min shaking at room temperature), the nanocomposite achieved a near-quantitative adsorption capacity in the first cycle (99–100%) and maintained a high level of removal efficiency in subsequent cycles with only a slight drop in efficiency at the fifth cycle (97.2%). The high level of desorption efficiency achieved using 0.10 M NaOH (10 min) also suggests that the high amount of arsenate adsorbed can be efficiently stripped under basic conditions.

The robust cyclic performance indicates that the primary adsorption mechanism is stable and can be largely reversed by the adopted desorption method. NaOH enhances the deprotonation of surface hydroxyl groups and enhances the competition of OH^−^, which helps to favor the removal of arsenate from metal-mediated binding sites (Zr–OH/Cu–OH domains) and destabilize the surface complexes through ligand exchange reactions. This is expected for inner-sphere adsorption at Zr-based nodes of UiO-66-NH_2_ (Zr–O–As bonding), as well as additional complexation at hydroxylated Cu sites, which are strong enough to provide high adsorption capacity at near-neutral pH conditions but can be partly reversed in basic solutions because of hydroxide-mediated competition and surface speciation. The good correlation between adsorption and desorption capacities in each cycle indicates that the active sites are still accessible and that there is no appreciable framework collapse or irreversible pore blocking during the operation.

The small efficiency loss with repeated use is what might be expected for nanocomposite sorbents and is probably due to a combination of factors: incomplete filling of the strongest binding sites by a remnant arsenate that is not completely desorbed, surface fouling by precipitated salts or strongly bound species, and a small amount of aggregation or compaction that may decrease the accessible surface area. However, the fact that the change is small (only a few percentage points over five cycles) suggests that these processes are not significant and that the Cu/UiO-66-NH_2_@GO system is chemically stable under both adsorption (near-neutral pH) and regeneration (basic NaOH solution) conditions. This is likely due to the natural stability of the UiO-66 framework, the role of GO in preventing particle agglomeration, and the immobilization of Cu nanoparticles in the nanocomposite, which work together to maintain reactive metal hydroxo sites and mass transport pathways.

In summary, the cycling results demonstrate the ability of the cycling data to verify that Cu/UiO-66-NH_2_@GO can be quickly regenerated and reused multiple times with high As(V) removal efficiency, which is an important criterion for cost-effective groundwater treatment. Although the current data set (five cycles) provides sufficient evidence of the promising reusability, further studies with more cycles and higher numbers of replicates, as well as more realistic water conditions (e.g., the presence of competing anions and natural organic matter), would be helpful.

### 3.6. Sorption Isotherms

The adsorption isotherm analysis gives a quantitative insight into the distribution of As(V) between the aqueous solution and the Cu/UiO-66-NH_2_@GO surface at 25 °C and hence reflects the nanocomposite’s affinity, surface heterogeneity, and saturation behavior with increasing driving concentration. The equilibrium data were fitted using the Langmuir and Freundlich equations (Table 2). The Langmuir equation models adsorption on a fixed number of identical sites with monolayer capacity [58], whereas the Freundlich equation is an empirical equation that models surface heterogeneity and site energy distribution [59]. The Langmuir equation has a higher goodness of fit (*R*^2^ = 0.960), compared to the Freundlich equation (*R*^2^ = 0.899), which indicates that the monolayer adsorption capacity on a fixed number of dominant sites is a better representation of As(V) adsorption on Cu/UiO-66-NH_2_@GO under the current experimental conditions.

Based on the Langmuir parameters (Table 2), the maximum monolayer capacity is high (*q*max = 747.9 mg g^−1^) with an affinity constant of *K*_L_ = 0.0583 L mg^−1^, which clearly indicates strong binding and high site availability. At the same time, the Freundlich constants (*K*_*F*_ = 67.14 and *n* = 1.75) also indicate favorable adsorption (*n* > 1) and the presence of a distribution of binding energies, which is quite reasonable for a multicomponent nanocomposite system where different types of functional sites are present.

From a mechanistic perspective, the affinity for Langmuir-like adsorption can be attributed to the presence of an abundance of defined high-affinity binding motifs that get saturated at higher loadings. In the case of Cu/UiO-66-NH_2_@GO, these motifs would include (i) the coordinatively active sites of the supported Cu domains that are capable of forming strong inner-sphere complexes with arsenate species; (ii) the UiO-66 framework nodes and amino-functional environment that are capable of facilitating electrostatic attraction and ligand exchange interactions depending on the solution chemistry; and (iii) the oxygenated functionalities of GO that improve dispersion and accessibility of the metal/MOF domains while also providing additional binding sites. Nanocomposite architecture thus supports efficient site occupation at low concentrations (high affinity) and a capacity ceiling at higher concentrations (site-limited saturation), as observed in the isotherm profile and supported by the model parameters obtained. From the isotherm data, Cu/UiO-66-NH_2_@GO has high affinity and capacity for As(V) at 25 °C, and the equilibrium data are best described by the Langmuir model (monolayer adsorption), although the Freundlich model is still valid for moderate surface heterogeneity.

### 3.7. Adsorption Mechanism

**Figure 8:**
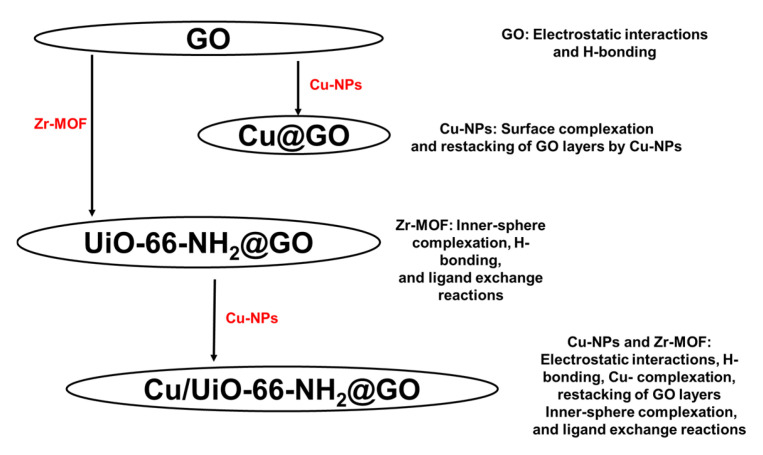
A schematic illustration showing the individual and synergistic contributions of GO, UiO-66-NH_2_, and Cu NPs.

The arsenate removal process from aqueous solutions by the Cu/UiO-66-NH_2_@GO nanocomposite is reasonably ascribed to the synergistic action of surface complexation, electrostatic attraction, H-bonding and ligand-assisted binding, as displayed in Figure 8. In aqueous solution, UiO-66-NH_2_ offers a high density of accessible Zr-based hydroxylated nodes (≡Zr–OH) that can form inner-sphere complexes with arsenate via ligand exchange reactions, forming ≡Zr–O–As bonds, especially in near-neutral pH environments where arsenate is predominantly present as H_2_AsO_4_^−^/HAsO_4_^2−^. At the same time, the added Cu NPs offer additional reactive sites; partial hydrolysis/oxidation reactions may generate Cu–OH/Cu–O moieties that can bind arsenate via bidentate/bridging Cu–O–As surface complexes and, under conditions of high local arsenate concentration, may facilitate the formation of sparingly soluble Cu-arsenate surface precipitates. Also, Cu ions possess a strong tendency to form inner-sphere complexes with oxyanions such as arsenate (AsO_4_^3−^), which enhances specific adsorption through chemisorption mechanisms. In addition, Cu sites can act as effective Lewis’s acid centers, promoting electrostatic attraction and coordination interactions with negatively charged arsenate species, particularly under environmentally relevant pH conditions.

Compared with other metals, Cu offers a balanced combination of high affinity, chemical stability, cost-effectiveness, and ease of incorporation into the UiO-66-NH_2_ framework without significantly disrupting its crystallinity. The incorporation of Cu onto the GO-supported UiO-66-NH_2_ also enhances electron transfer and the dispersion of active sites, further improving adsorption performance [60,61,62].

GO serves a dual function: its oxygenated functional groups (carboxyl, hydroxyl, epoxide) improve dispersion and suppress particle aggregation, simultaneously offering negatively charged domains that modulate interfacial hydration and facilitate mass transport of oxyanions to the metal centers. The amine groups of UiO-66-NH_2_ may also enhance binding by increasing hydrophilicity and generating localized regions of positive polarization (or protonated –NH_3_^+^ groups at lower pH values) that pre-concentrate arsenate near the framework surface, thus favoring inner-sphere coordination at Zr and Cu sites.

In general, the arsenate adsorption can be best explained by a cooperative process where GO facilitates the high accessibility of active sites, and Zr-oxo nodes and Cu-functionalities serve as the main binding sites through strong inner-sphere complexation and surface precipitation, despite the presence of competing groundwater ions. The As(V) sorption onto Cu/UiO-66-NH_2_@GO nanocomposite was found to be confirmed by FTIR spectroscopy, and the results are presented in Figure S1. The FTIR spectrum showed a strong peak at 873 cm^−1^, which is attributed to the As-O-Zr group [63], demonstrating that As(V) has been successfully bound to the Cu/UiO-66-NH_2_@GO nanosorbent. This proves the significance of coordinated interactions in As(V) sorption by showing that arsenate is bound inside the UiO-66-NH_2_ framework through the development of the As-O-Zr coordination bond.

### 3.8. Performance of Cu/UiO-66-NH_2_@GO for As(V) Removal from Environmental Water Samples

The applicability of Cu/UiO-66-NH_2_@GO for As(V) removal from real water samples was demonstrated by calibration performance, recovery, and precision in various matrices. One-way ANOVA revealed the absence of significant slope differences between the matrix-matched calibration and solvent-based calibration (*p* > 0.05), thus confirming that matrix effects were negligible and that external calibration can be safely used. The method was also validated using representative groundwater (two sources), tap water, and bottled water samples. As shown in Table 3, the background As(V) concentrations in the unspiked samples were low, ranging from ND to 0.033 mg L^−1^. After spiking at three concentration levels (3.0–3.5, 7.0–7.4, and 10.0–10.5 mg L^−1^), high recoveries were obtained for all water samples (99.5–100.0%) with good precision (RSD = 2.2–3.6%). Notably, this was achieved over the native sample pH range (6.75–8.15), thus demonstrating excellent As(V) extraction/adsorption performance under realistic water conditions. In summary, the data presented herein validate that Cu/UiO-66-NH_2_@GO is a precise, reproducible, and matrix-independent material for the efficient removal of As(V) from complex water samples.

## 4. Conclusions

In this work, a graphene oxide (GO)-based Cu/UiO-66-NH_2_ nanocomposite (Cu/UiO-66-NH_2_@GO) was developed and applied as a superior nanosorbent for the selective and rapid removal of As(V) in groundwater-mimicking matrices. In contrast with typical MOF-based adsorbents without a water-stable structure or the MOF particle agglomeration phenomenon, the combination of UiO-66-NH_2_ with the GO matrix created a stable 3D architecture where the active Zr-based nodes are still highly exposed to the environment, and the GO inhibits the agglomeration of particles and improves the diffusion of molecules. In addition to increasing the surface with more active sites, the addition of Cu-NPs produced a synergistic system with improved dispersion and mass transfer as well as a high density of adsorption sites. Consequently, the nanocomposite demonstrated ultrafast adsorption and reached equilibrium in 10 min, showing efficient removal over a broad pH range (2–11) and applicability to treat different types of groundwater. The study of the mechanism reveals that arsenate sequestration is driven by strong inner-sphere complexation via ligand exchange at coordinatively unsaturated zirconium centers, resulting in the formation of stable Zr-O-As bonds, and such a mechanism is supported by spectroscopic signals (XPS and Raman). In the meantime, electrostatic attraction and ligand-assisted interactions (both promoted by amino functionality and GO surface chemistry) enable the preconcentration of oxyanions in the vicinity of the active sites, which results in a faster overall adsorption process. Notably, the ternary architecture provides a feasible strategy for high-performance Zr-MOF-derived materials by integrating structural integrity and site accessibility with multifunctional adsorption moieties. Together, these findings show that Cu/UiO-66-NH_2_@GO is a unique water-stable, high-rate nanosorbent for arsenate and provide specific recommendations for the development of next-generation hybrid materials for the removal of arsenic from actual aqueous solutions.

## Figures and Tables

**Table 1:** Linear kinetic equations and sorption isotherm equations for the adsorption of As(V) on the Cu/UiO-66-NH_2_@GO nanocomposite at 25.0 °C, pH 6.8 and a 25 mg mass dose [25,26,27,28,29,30].

Kinetic Model	Linear Kinetic Equation	Calculated Parameters
PFO	$\ln\left(q_{e}-q_{t}\right)=\ln\;q_{e}-k_{1}t$	*q*_e_ (mg g^−1^) = 7.97
		*k*_1_ (min^−1^) = 0.220
		*R*^2^ = 0.638
PSO	$\frac{t}{q_{t}}=\;\frac{1}{k_{2}q_{e}^{2}}+\frac{t}{q_{e}}$	*q*_e_ (mg g^−1^) = 8.74
		*k*_2_(g mg^−1^ min^−1^) = 0.045
		*R*^2^ = 0.992
Elovich	$q_{t}=\frac{1}{\beta }(\ln\;\alpha \beta )+\frac{1}{\beta }\ln\;t$	*α* (mg g^−1^ min^−1^) = 6.79
		*β* (g mg^−1^) = 0.534
		*R*^2^ = 0.833

*q*_e_ and *q*_t_ (mg g^−1^) are the equilibrium adsorption capacity at equilibrium and at time *t*, respectively. *C*_e_ is the metal ion concentration at equilibrium. *K*_1_ and *K*_2_ are the first- and second-order adsorption rate constants of kinetic models. *α* (mg g^−1^ min^−1^) refers to the initial adsorption rate. *β* (g mg^−1^) is the desorption/activation parameter.

**Table 2:** The fitting parameters and linear adsorption isotherm equations for As(V) adsorption onto the Cu/UiO-66-NH_2_@GO nanocomposite at pH 6.8, a mass dosage of 25.0 mg, a contact period of 10 min and 25.0 °C.

Adsorption Isotherm Model/ Linear Equation	Isotherm Parameters
*Langmuir model*	
$\frac{1}{q_{e}}\;=\;\frac{1}{q_{m}}\;+\;\frac{1}{K_{L}\;q_{m}\;C_{e}}$	*q*_m_ (mg g^−1^) = 747.9
	*K*_L_ (L mg^−1^) = 0.0583
	*R*^2^ = 0.9604
*Freundlich model*	
$\ln q_{e}\;=\;\ln K_{F}\;+\;\frac{1}{n}\;\ln C_{e}$	*K*_F_ (L mg^−1^) = 67.14
	*n* = 1.748
	*R*^2^ = 0.8989

**Table 3:** Adsorptive removal performance of As(V) from spiked environmental water matrices using the Cu/UiO-66-NH_2_@GO nanocomposite.

Water Sample	pH	Spiked Conc. (mg L^−1^)	Detected Conc. (mg L^−1^)	%Recovery RSD ^b^
Groundwater (1)	6.83	3.5	0.018	99.5 ± 3.6
		7.2	0.011	99.8 ± 3.5
		10.3	0.033	99.7 ± 2.5
Groundwater (2)	6.75	3.0	0.015	99.5 ± 3.6
		7.0	0.021	99.7 ± 3.5
		10.0	0.013	99.9 ± 3.6
Tap water	7.65	3.2	ND	100.0 ± 3.2
		7.4	0.012	99.8 ± 3.1
		10.1	0.013	99.8 ± 2.8
Bottled water	8.15	3.0	0.017	100.0 ± 3.5
		7.1	ND	100.0 ± 2.2
		10.5	ND	100.0 ± 2.5

## Data Availability

The original contributions presented in this study are included in the article/Supplementary Material. Further inquiries can be directed to the corresponding author(s).

## Supplementary Materials

The following supporting information can be downloaded at: https://www.mdpi.com/article/10.3390/nano16080462/s1, Figure S1: FTIR spectra of Cu/UiO-66-NH2 (Zr)@ @GO nanocomposite before and after As(V) adsorption; Figure S2: XRD spectra of Cu/UiO-66-NH2 (Zr)@ @GO nanocomposite before and after As(V) adsorption.
