# Supplementary material for: Environmental Remediation of Arsenate-Contaminated Groundwater Using a Graphene Oxide-Supported Cu-NPs/UiO-66(Zr)-NH2 Nanocomposite
Source: Nanomaterials (Basel). 2026 Apr 14;16(8):462. doi: 10.3390/nano16080462 (PMC13119152; doi:10.3390/nano16080462)
Supplement: Supplementary file 1 [file nanomaterials-16-00462-s001.zip › nanomaterials-4221148-supplementary.pdf]

Supplementary Materials:

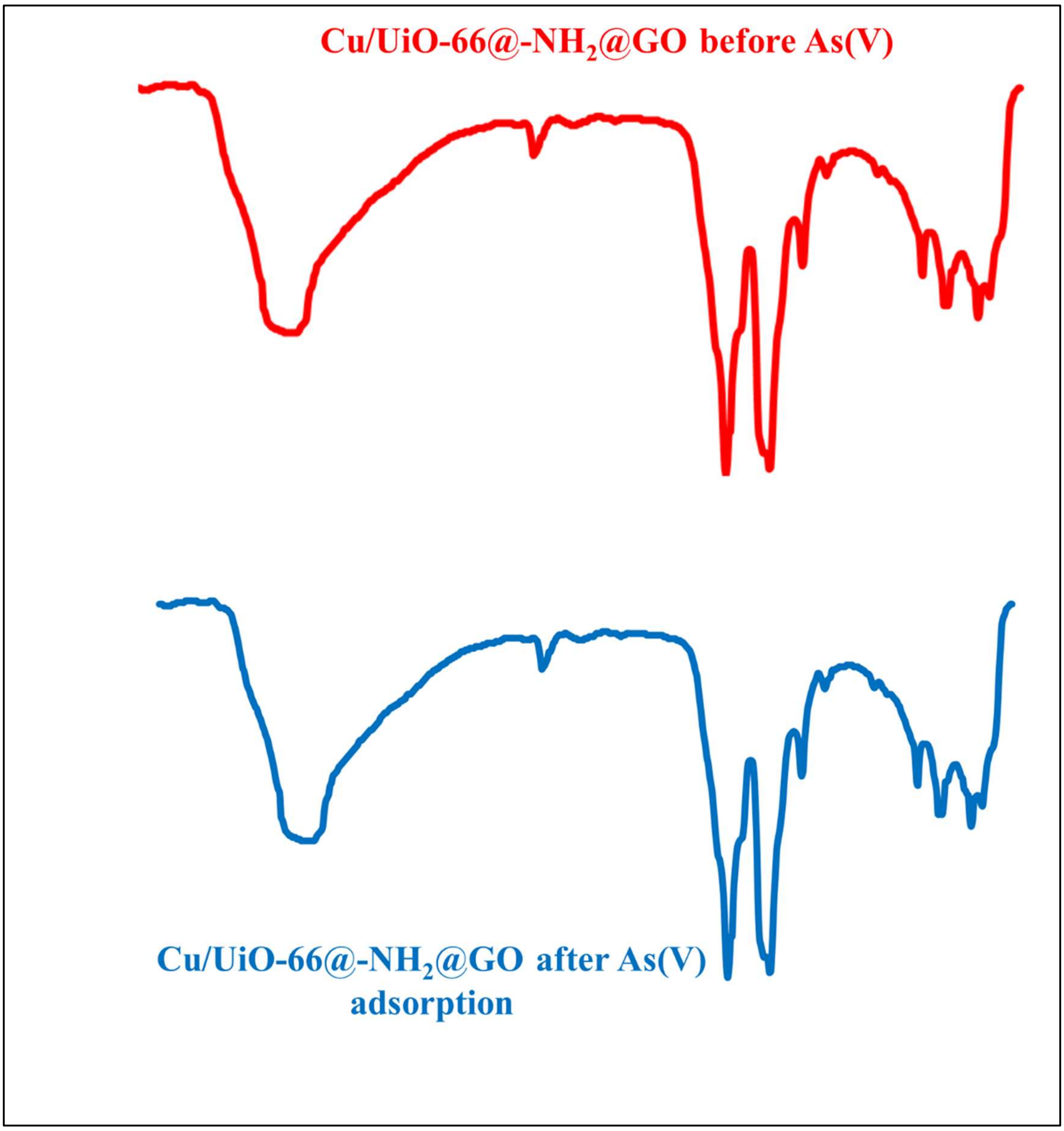

Figure S1: FTIR spectra of Cu/UiO-66-NH$_2$ (Zr)@ @GO nanocomposite before and after As(V) adsorption

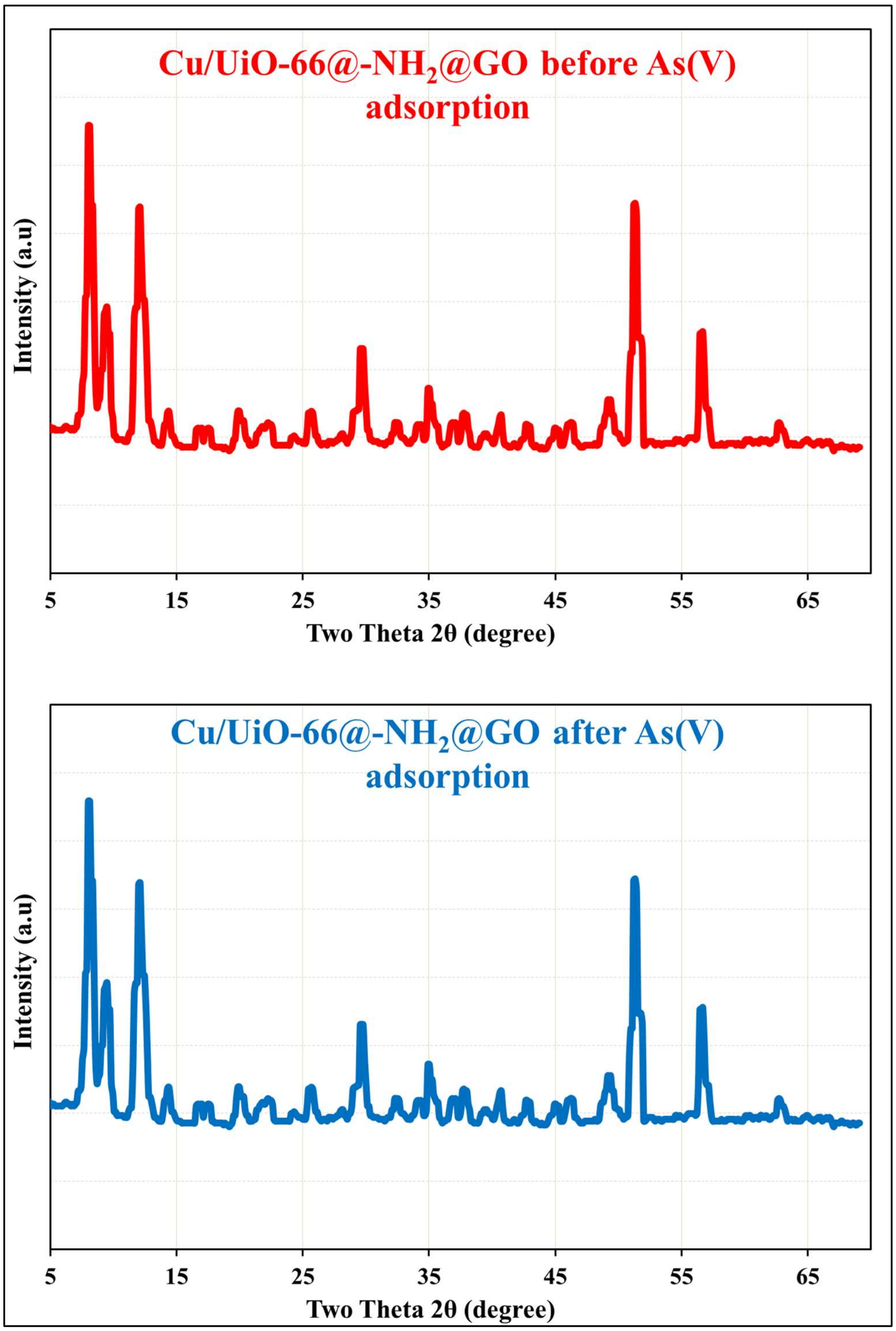

Figure S2: XRD spectra of Cu/UiO-66-NH_2 (Zr)@ @GO nanocomposite before and after As(V) adsorption
